# Potential Roles and Mechanisms of Curcumin and its Derivatives in the Regulation of Ferroptosis

**DOI:** 10.7150/ijbs.90798

**Published:** 2024-09-09

**Authors:** Yuan Zhang, Chenghao Yu, Cheng Peng, Fu Peng

**Affiliations:** 1 State Key Laboratory of Southwestern Chinese Medicine Resources, Chengdu University of Traditional Chinese Medicine, Chengdu 611137, China.; 2 Department of Pharmacology, Key Laboratory of Drug-Targeting and Drug Delivery System of the Education Ministry, Sichuan Engineering Laboratory for Plant-Sourced Drug and Sichuan Research Center for Drug Precision Industrial Technology, West China School of Pharmacy, Sichuan University, Chengdu 610041, China.

**Keywords:** Curcumin, Ferroptosis, Iron, Oxidation, Anticancer effect, Organ protective effect

## Abstract

Ferroptosis is a recently discovered iron-dependent mode of oxidatively regulated cell death. It is not only associated with a wide range of diseases, but it is also a key component of many signaling pathways. In general, ferroptosis is a double-edged sword. On one hand, it induces nonapoptotic destruction of cancer cells, but on the other, it may lead to organ damage. Therefore, ferroptosis can be drug-targeted as a novel means of therapy. The properties of curcumin have been known for many years. It has a positive impact on the treatment of diseases such as cancer and inflammation. In this review, we focus on the regulation of ferroptosis by curcumin and its derivatives and review the main mechanisms by which curcumin affects ferroptosis. In conclusion, curcumin is a ferroptosis inducer with excellent anticancer efficacy, although it also exhibits organ protective and reparative effects by acting as a ferroptosis inhibitor. The differential regulation of ferroptosis by curcumin may be related to dose and cell type.

## 1. Introduction

**Figure 1:**
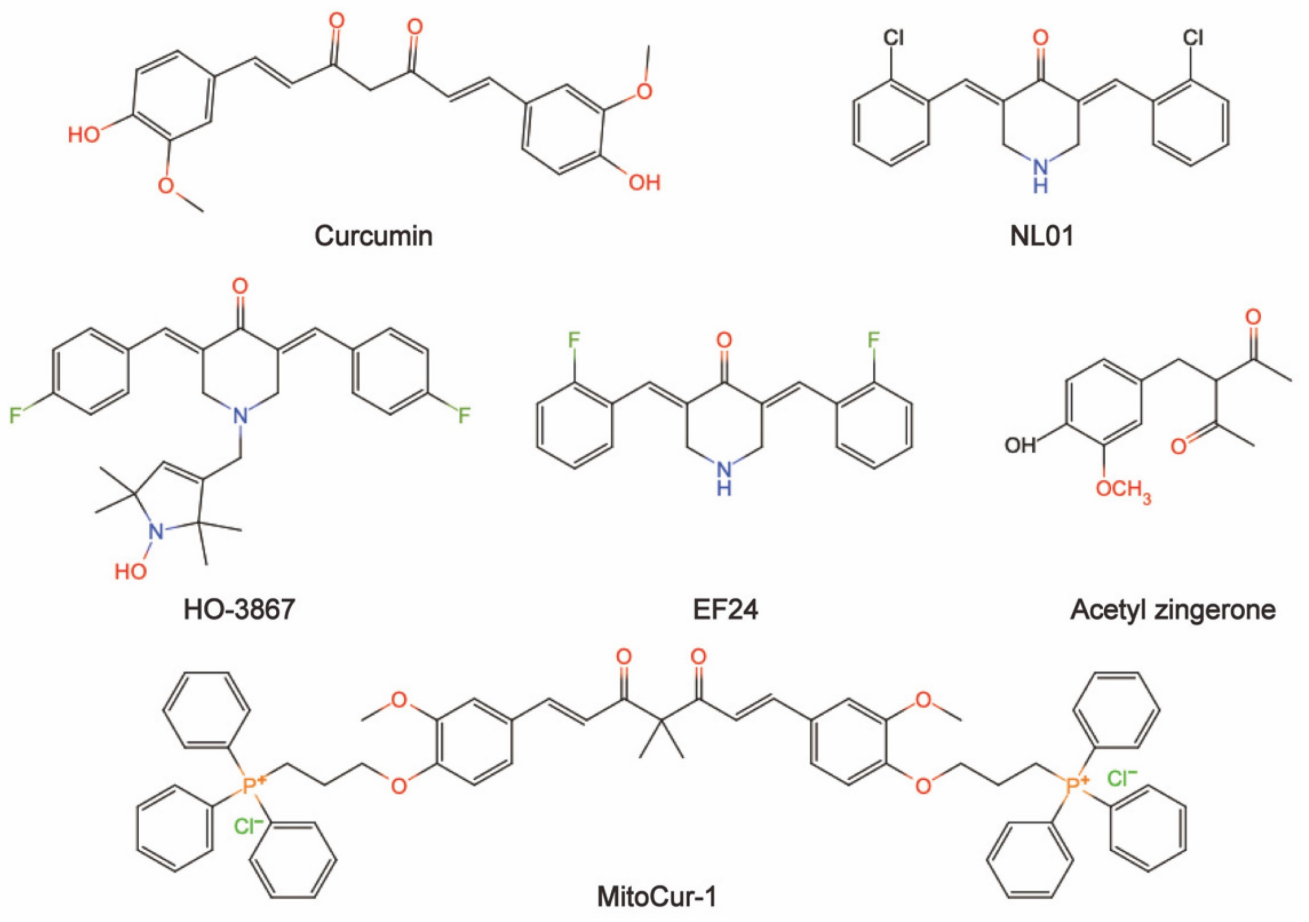
The chemical structures of curcumin and some of its derivatives (drawing by InDraw).

**Figure 2:**
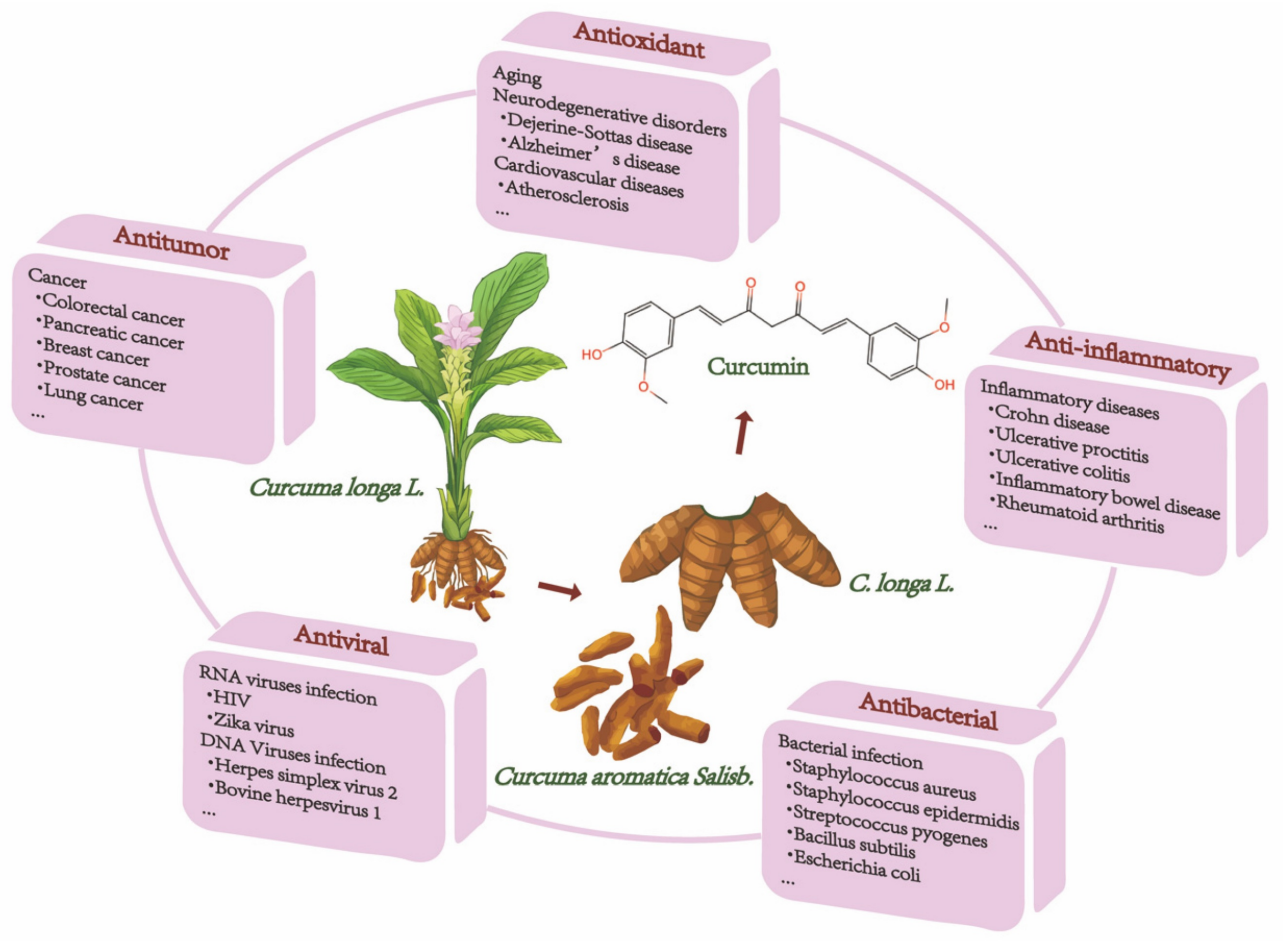
The sources and biological activities of curcumin.

Natural ingredients extracted from traditional Chinese medicine (TCM) are immeasurable and have been used clinically for the treatment of various diseases^1^,^2^. Curcumin is among these molecules. Historically, curcumin was isolated in 1815 by Vogel and Pelletier and was derived from the root tuber and the rhizome of *Curcuma longa L.*^3^. This herb has traditionally been known to improve blood circulation, eliminate blood stasis, and relieve pain, an effect also attributed to the presence of its phenolic constituent curcumin^4^. Modern studies have shown that curcumin has a wide range of pharmacological activities, such as antioxidant, antitumor, anti-inflammatory, and antiviral properties and has shown promising therapeutic potential in preclinical and clinical studies^5^-^7^. Moreover, curcumin is being "Generally Recognized as Safe" by the US Food and Drug Administration (FDA)^8^. Chemically, curcumin is insoluble in water and is soluble in organic solvents such as acetic acid, ketone, alkali and chloroform. Due to its hydrophobicity, instability, rapid metabolism in vivo, and poor intestinal absorption^9^,^10^, curcumin has inherent drawbacks, such as its low bioavailability, poor pharmacokinetic/pharmacodynamic properties, and poor efficacy in certain disease models^11^-^13^. Several efforts have been made to improve these properties. First, it is worthwhile to combine curcumin with photodynamic therapy. Recent findings have indicated that curcumin combined with photodynamic therapy prolonged the action time and increased the bioavailability of curcumin, resulting in a more efficient effect on cancer with a broad spectrum of targets^3^. Second, curcumin encapsulation using special polymers, such as liposomes and nanomaterials, has the advantages of high drug loading, high encapsulation rate, and high safety to improve curcumin bioavailability^14^-^16^. Furthermore, synthesized derivatives that are structurally similar to curcumin can be targeted to overcome these limitations and achieve good therapeutic prospects^17^. The chemical structures and other chemical information of curcumin and some of its derivatives are summarized in Fig. 1 and Table 1. The sources and biological activities of curcumin are displayed in Fig. 2.

Dixon first introduced the concept of ferroptosis, a new modality of cell death, to the world in 2012^18^. Since then, research on ferroptosis has been growing exponentially over the past few years. Unlike apoptosis, autophagy, and necroptosis, ferroptosis is a distinctive programmed cell death mechanism^19^, as well as a new type of oxidatively regulated cell death driven by iron-dependent lipid peroxidation^20^,^21^. Specifically, when intracellular levels of lipid reactive oxygen species (L-ROS) exceed the antioxidant activity of glutathione peroxidase 4 (GPX4), this leads to a breakdown of cellular redox homeostasis^22^. Interestingly, ferroptosis appears to be more of a cellular "sabotage" than an active "suicide"^23^. In other words, ferroptosis refers to an iron-dependent, oxidative form of non-apoptotic cell death. Unlike apoptosis or autophagy, which appears to occur as a consequence of specialized molecular events taken on the initiative of cells for altruistic benefit, ferroptosis can be triggered by the depletion of the amino acid cysteine or the inhibition of GPX4, which is associated with the consumption of ATP or the production of lipid hydroperoxides with cell destruction, leading to catastrophic damage^24^. Mitochondria are the main intracellular generators of reactive oxygen species (ROS)^25^, and the focal point of iron metabolism and homeostasis^26^. Ferroptotic cells show characteristic morphological changes including reduction in mitochondrial volume, decrease or even disappearance of mitochondrial crista, increase in the density of the mitochondrial membrane, and rupture of outer mitochondrial membranes^27^,^28^.

Ferroptosis can be triggered by a variety of physiological conditions and pathological stresses in humans and animals and is increasingly recognized as an adaptive feature for the elimination of malignant cells^18^. Experimental compounds or drugs are capable of inhibiting ferroptosis in both cancer cells and certain normal cells^29^-^31^. Meanwhile, activation of mitochondrial voltage-dependent anion channels and mitogen-activated protein kinases, up-regulation of endoplasmic reticulum stress, and inhibition of cystine/glutamate resistant formate play key roles in the induction of ferroptosis^32^. Surprisingly, treatment-resistant cancer cells, especially those in a mesenchymal state and prone to metastasis, are highly susceptible to ferroptosis^33^. It is critical for suppressing tumorigenesis by removing cells that are deficient in key nutrients in the environment or damaged by infection or environmental stress^34^. In addition, many organ injuries and degenerative diseases are driven by ferroptosis^35^-^37^. Thus, pharmacological modulation of ferroptosis, through its induction and inhibition, has great potential in the treatment of drug-resistant cancers, ischemic organ damage, and other degenerative diseases associated with lipid peroxidation.

The key to ferroptosis is the iron-catalyzed peroxidation of polyunsaturated fatty acid (PUFA)-containing phospholipids (PUFA-PLs), which exceeds the buffering capacity of the defense system and ultimately leads to cell death. Accumulated intracellular iron is the trigger for ferroptosis as it can produce highly reactive free radicals via the Fenton reaction, which can lead to ferroptosis^38^. It has been shown that elevated intracellular ROS due to oxidative stress can accelerate the onset of ferroptosis or increase cellular susceptibility to ferroptosis inducers^39^. Intracellular iron accumulation and oxidation are the two central biochemical events causing ferroptosis. The ability of curcumin to chelate iron and regulate oxidation predicts that curcumin may have a role in regulating ferroptosis, which was confirmed in recent studies^40^,^41^. Herein, we review these activities and analyze the possible underlying mechanisms involved, for which there has been little information to date, in the hope of informing and contributing to the screening of natural medicines.

## 2. Curcumin and its derivatives promoted ferroptosis in cancerous cells

### 2.1 Regulation of iron metabolism

Iron's role in the redox cycle where it can act as an electron carrier allows it to catalyze the Fenton reaction with H_2_O_2_, followed by the production of highly reactive hydroxyl radicals (•OH), which, as part of ROS, can damage macromolecules such as lipids, proteins, and DNA, as well as cellular organelles such as lysosomes and mitochondria, and has been linked to various signaling pathway impairments^42^. Iron homeostasis is tightly regulated in healthy cells to balance systemic absorption and distribution as well as cellular uptake, storage and export^43^,^44^. A distinctive hallmark of cancer is dysregulation of iron homeostasis, including overexpression of genes involved in iron metabolism and increased intracellular labile iron^45^,^46^. Of particular note, tumor cell growth and survival cannot occur without an increase in iron concentration, and an increase in intracellular labile iron pools is also essential for cancer cell metastasis. However, increased iron levels can also lead to ferroptosis via the Fenton reaction^38^. Cancer management is becoming as diverse as the disease itself. Targeting iron metabolism in cancer cells is a powerful and promising therapeutic area.

Cellular iron metabolism involves the regulation of labile iron in the cell membrane, which is a minor but vital part of the total amount of redox-active iron in the cell^19^. Convincing evidence has shown that curcumin and its derivatives can induce iron accumulation by increasing the concentration of Fe^2+^ in a wide range of tumor cells and tissues^47^-^51^. Furthermore, Yin et al. developed a cascade catalytic nanoplatform (CaO_2_/Tf /CUR) for ion interference therapy. CaO_2_/Tf/CUR with tumor-targeting action was internalized in tumor cells and decomposed to release Ca^2+^ and curcumin. CaO2/Tf/CUR activated the mitochondrial apoptotic signaling pathway by inducing Ca^2+^ overload which further led to cellular damage. Conversely, the generated H_2_O_2_ disrupted the structure of transferrin (TF) thus releasing Fe^3+^. Ferroptosis is triggered by conversion to hydroxyl radicals via trivalent iron ion-mediated Fenton reaction^52^. DMT1 acts as a proton pump and utilizes the cell membrane potential for active iron transport. Ling et al. found that HO-3867 was able to upregulate DMT1 expression by regulating the level of p53^51^.

Iron uptake through the transferrin receptor 1 (TFR1) and storage in ferritin (FT) is vital for regulating the labile iron pool in the cytoplasm. Among the many factors that affect iron metabolism, FT has been investigated to understand the mechanisms of release/accumulation of reactive iron as an important regulatory point for iron metabolism homeostasis and ferroptosis^53^. Activation of iron metabolism-related proteins promotes ferroptosis^54^. However, another argument suggests that the regulation of labile iron by overexpression of FT decreases the production of reactive free radicals, whereas the down-regulation of FT increases oxidative damage^55^. Curcumin has been proven to modulate TFR1 levels, as well as the two subunits of FT, in cancer cells, ferritin heavy chain (FTH1) and ferritin light chain (FTL), which in turn influences intracellular iron transport and storage functions and alters labile iron levels^50^,^56^-^58^. The suppression of IREB2, a prominent transcription factor involved in the regulation of iron metabolism, resulted in a notable increase in the expression of FTL and FTH1, consequently preventing the occurrence of erastin-induced ferroptosis^59^. Meanwhile, siIREB2 interference could reduce curcumin-induced cell death in A549 and H1299 cells, indirectly indicating that curcumin could participate in the ferroptosis process in lung cancer cells by targeting IREB2. Furthermore, curcumin can down-regulate the level of NCOA4 in clear cell renal cell carcinoma by upregulating the ADAMTS18 gene, which contributes to cell death and reverses resistance to sunitinib. This is because the delivery of FT to lysosomes requires NCOA4, which is highly aggregated in autophagosomes. In other words, cells lacking NCOA4 do not degrade FT, leading to a decrease in bioavailable intracellular iron^60^.

The enzyme known as heme oxygenase-1 (HO-1) converts heme into free iron, carbon monoxide, and bilirubin. Under typical physiological circumstances, HO-1 scavenges ROS and offers cellular defense^61^. Yet, curcumin-induced HO-1 overactivation raises intracellular ferric ions, MDA, and ROS levels in cancer cells, surpassing FT's buffering ability and leading to the uncontrollably high release of iron and the consequent disturbance of iron metabolism^48^,^56^,^58^,^62^,^63^. Remarkably, while inhibiting the expression of GPX4, curcumin not only directly activates HO-1, but also upregulates its expression by activating Nrf2, promoting ferroptosis^56^.

### 2.2 Inhibition of the antioxidant system

Organisms have developed a variety of antioxidant regulators, such as superoxide dismutase (SOD), catalase (CAT), and glutathione peroxidase (GPX), as a means to protect against oxidative damage^64^. However, when these defense mechanisms are disrupted, resulting in an imbalance between the production and removal of ROS, cells experience impaired functionality or even death due to oxidative stress^65^. Cancer cells exhibit intrinsic oxidative stress, which makes them more susceptible to further ROS production by pro-oxidant anticancer agents (PAAs). However, PPAs inevitably generate ROS in normal cells, resulting in a narrow therapeutic window and high toxicity, which greatly limits their clinical application^66^. In the tumor microenvironment (TME), tumor cells evolve to gradually be insensitive to oxidative stress or other harmful forces, leading to their resistance to stress-inducing agents such as chemotherapy and radiotherapy^67^. In this context, ferroptosis could be a robust tool for developing a novel strategy by amplifying oxidative stress or inhibiting antioxidant molecules in tumor cells.

#### 2.2.1 GPX4/GSH

Ferroptosis inducers such as glutamate and erastin can drain GSH and inactivate the enzyme activity of GPX4 by blocking the import of cystine by the cystine/glutamate antiporter (system Xc-)^68^,^69^. GPX4 is an essential regulator of ferroptosis in cancer cells as well as a key factor in maintaining cellular redox homeostasis^70^. GSH, as an important iron inhibitor and non-enzymatic antioxidant, provides an important defense system that protects cells from different types of oxidative stress^71^. GPX4 converts GSH to oxidized glutathione (GSSG) and the cytotoxicity of lipid peroxides (L-OOH) to the corresponding alcohol (L-OH)^72^-^74^. The ratio between GSH and GSSG generally indicates the level of cellular oxidative stress. However, L-OOHs are unstable and can be broken down into reactive compounds such as MDA, which acts as a "second messenger of oxidative stress" due to its long half-life and its ability to diffuse out of the formation site^75^,^76^. Together, GPX4 and GSH appear to be the major determinants of the balance between cell proliferation and death. Inactivation of GPX4 or depletion of GSH in cells may lead to ferroptosis^77^. ALZ003-induced AR ubiquitination improves glioblastoma resistance to temozolomide by disrupting GPX4-mediated redox homeostasis and promoting subsequent ferroptosis^78^. Curcumin inhibits GPX4 levels by upregulating Nrf2 expression, triggering the molecular and cytological characteristics of ferroptosis in breast cancer cells^56^. Curcumin and its derivative EF24 repress GPX4 expression and increase MDA and ROS levels to exert a pro-ferroptosis effect in osteosarcoma cells^48^,^79^. Similarly, a notable increase in MDA content was detected in tumor tissues of non-small cell lung carcinoma mice treated with curcumin, accompanied by a decrease in SOD activity^49^. It is well known that SOD is an indispensable constituent of the antioxidant enzyme system in biological systems. Meanwhile, a reduction in GSH content and L-OOH levels was measured in curcumin-treated TNBC cells^58^. Liu et al. designed a hypoxia-responsive nanodelivery system based on angelica polysaccharides and used curcumin as a model drug. When such curcumin-loaded micelles were employed in hepatocellular carcinoma mice, ferroptosis in solid tumors could be selectively enhanced by reducing GSH under hypoxic conditions^80^. Zhong et al. constructed a photodynamic therapy/photothermal therapeutic system by loading curcumin onto Au NRs, taking advantage of the varying pH and ROS levels of tumors and normal tissues to promote the production of lipid peroxide in melanoma^81^.

#### 2.2.2 System Xc-

System Xc- consists of two subunits, SLC7A11 and SLC3A2, and is an amino acid reverse transporter protein. Cystine and glutamate are exchanged intracellularly and extracellularly through System Xc- in a 1:1 ratio^18^. Absorbed cystine is reduced in the cell to cysteine, which continues to be involved in GSH synthesis and influences GPX activity. Interestingly, curcumin induced a decrease in SLC7A11 levels in tumor tissues from homozygous Lewis lung carcinoma mice, and this phenomenon was similarly observed in several lung cancer cells^49^,^82^. Furthermore, recent studies found that curcumin also negatively regulated the expression of SLC7A11 in colorectal cancer cells through PI3K/Akt/mTOR and p53 signaling, which selectively caused ferroptosis and suppressed cancer cell proliferation^83^,^84^.

#### 2.2.3 Glutamine

Glutamine is a conditionally essential amino acid for rapidly proliferating tumor cells^85^. Studies have shown that aberrant glutamine metabolism can promote cellular ferroptosis by enhancing the accumulation of lipid peroxides^54^,^86^,^87^. curcumin facilitates glutamine consumption by upregulating the expression of solute carrier family 1 member 5 (SLC1A5), a critical glutamine transporter, and exerts its antitumor effects against breast cancer in vitro and in vivo^50^.

#### 2.2.4 FSP1-CoQ10- NAD(P)H

Several studies have revealed that inhibition of GPX4 does not initiate ferroptosis in some cancer cell lines, suggesting the existence of alternative antiferroptosis regulators in cancer cells^88^-^90^. In line with this hypothesis, recent studies have confirmed the presence of the FSP1-CoQ10- NAD(P)H pathway as an independent parallel system involved in the curbing of lipid peroxidation and ferroptosis in cooperation with GPX4/GSH^88^. FSP1, previously known as apoptosis-inducing factor mitochondria-associated protein 2 (AIFM2), was identified as a GPX4-independent ferroptosis inhibitory protein^89^. As a lipophilic free radical adsorbing antioxidant, FSP1 prevents the propagation of lipid peroxides. More specifically, inositolized FSP1 is recruited to plasma membranes and uses NAD(P)H to catalyze the reduction of ubiquinone (CoQ10), forming ubiquinol as a free radical trapping antioxidant to terminate serum lipid peroxidation (LPO) and ultimately inhibit ferroptosis^74^. Curcumin downregulates the levels of FSP1, CoQ10, and NAD+/NADH proteins in tumor cells. Meanwhile, the positive expression of FSP1 in tumor tissues was also obviously downregulated by curcumin. Further studies revealed that an inhibitor of ferroptosis (Fer-1) significantly suppressed these curcumin-mediated effects^91^. Pharmacological inhibition of FSP1 synergized with inhibition of GPX4 to induce ferroptosis in many cancer cells. Thus, dual repression of GPX4 and FSP1 by curcumin is considered promising cancer therapy^92^.

#### 2.2.5 Thioredoxin reductase

Thioredoxin reductase (TrxR) catalyzes the reduction of disulfide bonds in thioredoxin (Trx) with the help of NAD(P)H. Subsequently, Trx interacts with a series of downstream proteins through thiol-disulfide exchange to regulate redox signaling events and protect cells from ROS-induced oxidative damage^93^,^94^. Overall, TrxR, together with Trx and NAD(P)H, constitutes a sulfur-oxygen reduction protein system that maintains cellular redox homeostasis^95^. oxidative stress, cancer cells typically overexpress TrxR^96^, making the enzyme an attractive cancer-specific target^97^-^99^. The curcumin derivative 2c was able to selectively cause ROS-dependent apoptosis and ferroptosis in human non-small cell lung cancer cells, but not in human normal lung cells, by covalently modifying the Sec-498 residue of intracellular TrxR and generating ROS. Of interest, curcumin derivative 2c also dramatically arrested the growth of transplanted tumors in nude mice with non-small cell lung cancer cells without obvious toxicity to the liver or kidneys^66^.

### 2.3 Other mechanisms

Wang et al. validated in vivo that silencing of circFOXP1 enhanced the expression of ferroptosis markers, establishing elevated levels of circFOXP1 in tumors and supporting its potential prognostic role in lung cancer. The specific mechanism involved circFOXP1 enhancing SLC7A11 expression in cancer cells by direct sponge adsorption of miR-520a-5p. Curcumin and quercetin inhibited the expression of circFOXP1 in lung cancer cells by regulating the miR-520a-5p/SLC7A11 axis, which in turn affected cell growth, migration and invasion as well as ferroptosis^47^. Curcumin can induce ferroptosis in colorectal cancer cells by down-regulating JNK signaling^100^, as well as affecting a variety of ferroptosis-related genes^101^. MitoCur-1 reversed melanoma cell resistance to vemurafenib by inhibiting USP14 and promoting ferroptosis^102^. A growing number of findings suggest that ferroptosis frequently interferes with the immune response, leading to inflammation-associated immunosuppression^103^,^104^. During the development of alternative herbal medicines for the treatment of gastric cancer based on transcriptomic analysis of immune infiltration and ferroptosis, Li et al. discovered that TLR4 and KRAS, as common genes for immune infiltration and ferroptosis, play a major role in the progression of gastric cancer. Based on the prediction of these two key genes, several herbal components, including curcumin, provide research directions and alternative therapies for immunomodulation in the TME and ferroptosis of gastric cancer^105^. A recent study demonstrated that NL01 induced ferroptosis in two types of ovarian cancer cells. Intriguingly, this new derivative of curcumin was 13-times more potent than curcumin in curbing the growth of cancer cells. Further studies revealed that the mechanism by which NL01 contributes to ferroptosis is associated with lactate metabolism. It can reduce lactate uptake from the extracellular environment by decreasing the expression of hydroxycarboxylic acid receptor 1 (HCAR1)/monocarboxylic acid transporter protein 1 (MCT1), and activate the AMPK/ SREBP1 pathway to lower glucose uptake and lactate production to improve energy metabolism. Knockdown of HCAR1 expression revealed phenotypic and pathway alterations similar to those of NL01 treatment, which inversely validated the rationale for targeting lactate metabolism^106^.

**Figure 3:**
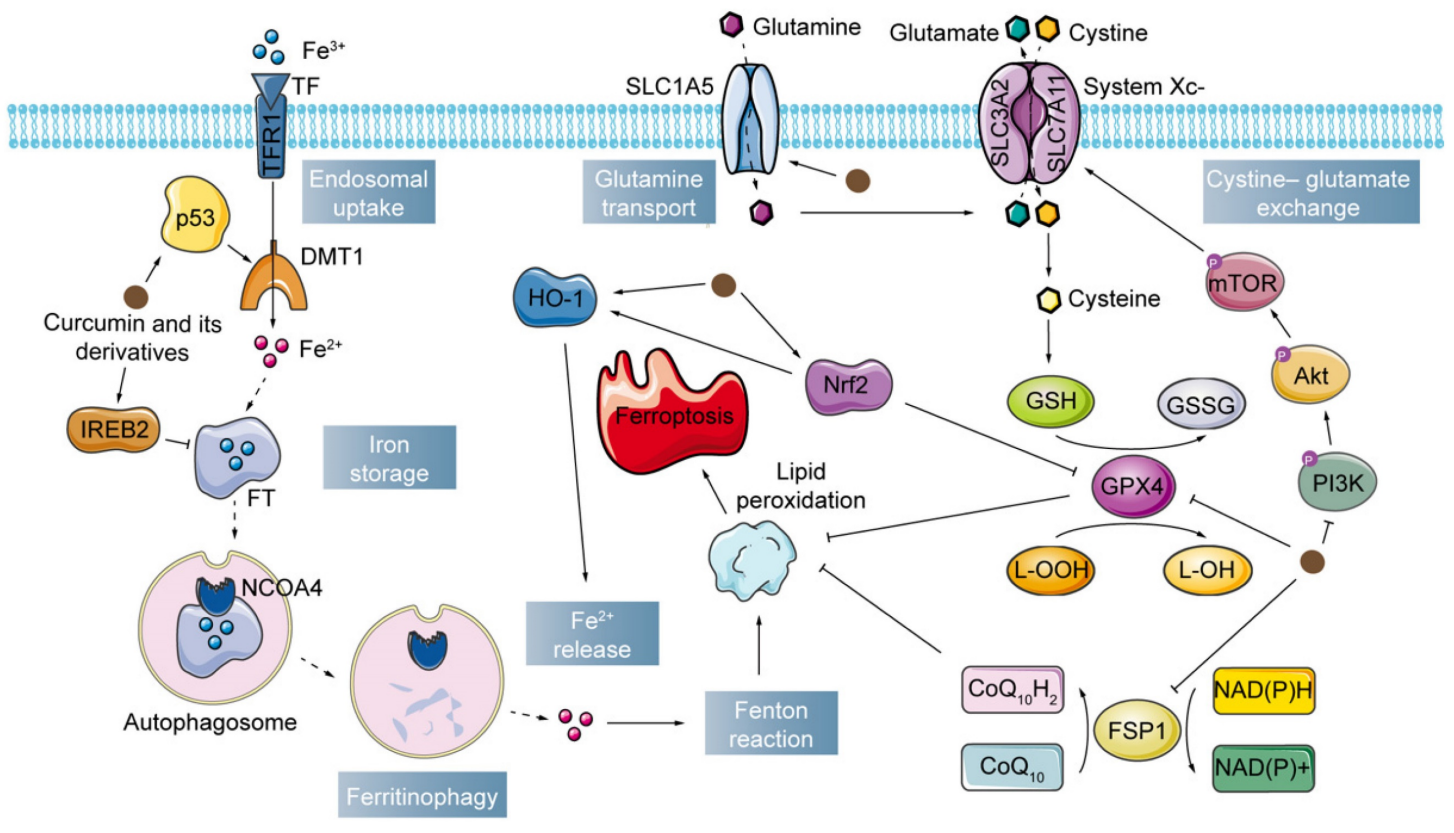
Curcumin and its derivatives exert antitumor effects by modulating the ferroptosis pathway.

Last but not least, curcumin also affects the expression of proteins related to endoplasmic reticulum stress and autophagy pathways in cancer cells undergoing ferroptosis^56^. It is reasonable to infer that endoplasmic reticulum stress and autophagy may also be involved in the modulation of ferroptosis in cancer cells by curcumin, which requires further experimental verification. The mechanism of action of curcumin and its derivatives in cancer cells is summarized in Fig. 3 and Table 2.

## 3. Curcumin and its derivatives inhibited ferroptosis in tissue-damaged models

### 3.1 Brain

Curcumin is one of the few polyphenols that exhibit dramatic protective effects against ferroptosis-induced damage to cells^107^. Through activation of the Nrf2/HO-1 pathway, curcumin can both restrict high glucose-induced neuronal (N2a) cell injury^108^ and promote clearance of intracranial hematomas, reduce perihematoma brain edema as well as promote neurological recovery after intracerebral hemorrhage (ICH)^109^. The main underlying mechanisms that produce this event are closely related to the antioxidant system and the iron metabolism regulatory system of curcumin. Curcumin pretreatment also effectively attenuated oxidative stress and neural ferroptosis in the ICH model by upregulating the antioxidant activity of mesenchymal stem cells (OM-MSCs)^110^. In addition, encapsulation of curcumin in nanoparticles (Cur-NPs) can better facilitate the delivery of curcumin to the brain through the physiological barrier^111^. Yoko et al. applied hybrid molecules consisting of the oxidized indole backbone of neuroprotective compounds and the polyphenol backbone of curcumin to mouse hippocampal HT22 cells for experimental purposes and noted that these preparations possessed superior neuroprotection and lower cytotoxicity compared to curcumin. In particular, they scavenge ROS to shield cells from endogenous oxidative stress as well as ferroptosis through stimulation of antioxidant-responsive elements and chelation of ferrous ions, and finally foster neuronal survival^112^-^115^.

### 3.2 Heart

Combining various modern techniques, Feng et al. identified the key gene TGFBR1 from the efficient screening of immunity and ferroptosis-related biomarkers and immunomodulatory ability of herbal ingredients. TGFBR1 was found to dock well with curcumin, which was further validated to substantially attenuate myocardial fibrosis for the management of valvular atrial fibrillation^116^. Diabetes disordered the arrangement of cardiomyocytes and significantly enlarged the degree of myocardial fibrosis and collagen expression in cardiomyocytes. Curcumin treatment increases the nuclear translocation of Nrf2 and the expression of GPX4 and HO-1, alleviates glucose-induced cardiomyocyte injury, and reverses erastin-induced ferroptosis in cardiomyocytes^117^. Furthermore, curcumin mitigates oxidative stress, ferroptosis, and liver, pancreas, and heart injury after myocardial ischemia-reperfusion injury by modulating cellular lipid composition^118^.

### 3.3 Liver

Curcumin can help promote the excretion of excess Cu^2+^ in a concentration-dependent manner, diminish the accumulation of Cu^2+^, reduce intracellular Cu^2+^ content in hepatolenticular degeneration (HLD) hepatocytes, and protect the copper-injured HLD model from oxidative stress based on the Nrf2/HO-1/GPX4 signaling pathway to achieve a protective function in normal rat hepatocytes^119^. Parallel to this, in liver-injured heterozygous silver crucian carp, curcumin relieved ammonia-induced oxidative stress and ferroptosis by inhibiting ROS and MDA levels along with activation of the Nrf2 pathway^120^.

### 3.4 Kidney

There is no specific treatment for kidney damage caused by rhabdomyolysis. Ferroptosis is involved in cellular wounding and inflammation induced by rhabdomyolysis in vivo and in vitro. Curcumin dampened the characteristic changes in ferroptosis, which subsequently improved renal injury and inflammation^41^,^121^. HO-1 is a key pathway involved in the protective properties of curcumin^122^. The hydrophobic core of ferritin nanocages can load curcumin and specifically deliver it to the site of renal injury, improving bioavailability. More importantly, curcumin and ferritin nanocages can synergize their antioxidant activities to reduce ferroptosis and invert the pathological process of ischemia-reperfusion acute kidney injury (IR-AKI) by reducing ROS and absorbing overloaded iron, respectively^123^.

### 3.5 Other properties

**Figure 4:**
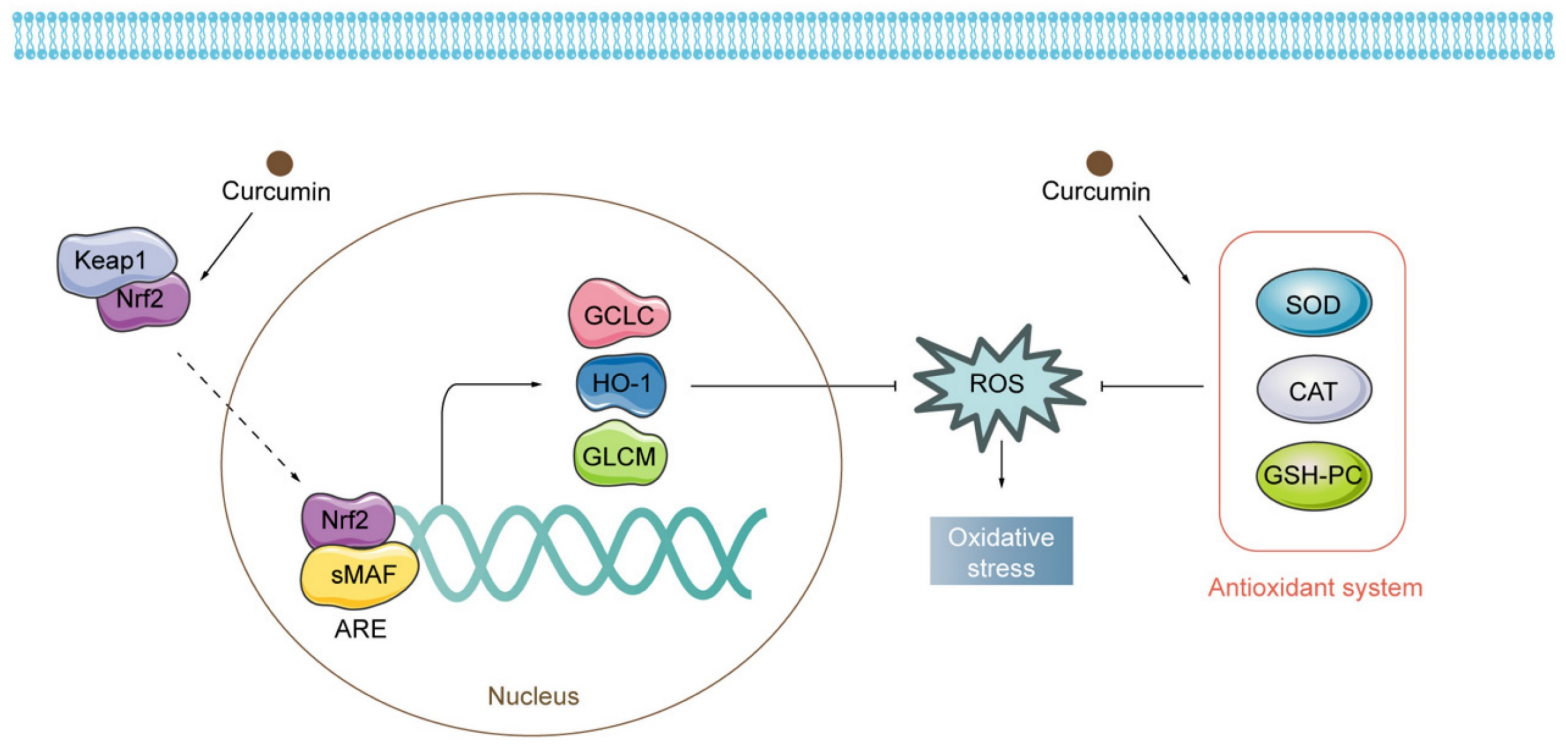
Regulatory mechanisms of curcumin on oxidative stress. Curcumin exerts antioxidant properties by activating both Nrf2-related pathways and the antioxidant system.

Curcumin improves functional and histological lung damage from cigarette smoke and eases pulmonary ferroptosis, suggesting that curcumin may play a beneficial role in patients with COPD by limiting ferroptosis^124^. Encapsulation of cerium oxide nanoparticles (CeO_2_) and curcumin in mannose-modified chitosan (MCS) enhanced the therapeutic efficacy of inflammatory bowel disease (IBD), on the one hand, by increasing the expression of GSH and GPX4 to protect intestinal cells from ferroptosis, and, on the other hand, it could leverage the targeting of macrophages to minimize effects beyond the site of colonic inflammation^125^. Recent studies have demonstrated this phenomenon of curcumin suppression of ferroptosis in a mouse model of periodontal tissue injury in periodontitis, in which lipid peroxidation and System Xc- are involved and exert a crucial role^126^. Curcumin and its derivative acetyl zingerone can ameliorate osteoarthritis (OA) via the Nrf2 pathway^127^,^128^. In testicular tissue, curcumin upregulated SP1 and PRDX6 to stimulate self-protection against damage from ferroptosis^129^. The efficacy of curcumin and its derivatives in tissue-damaged models is shown in Fig. 4 and Table 3.

## 4. Conclusions and perspectives

**Figure 5:**
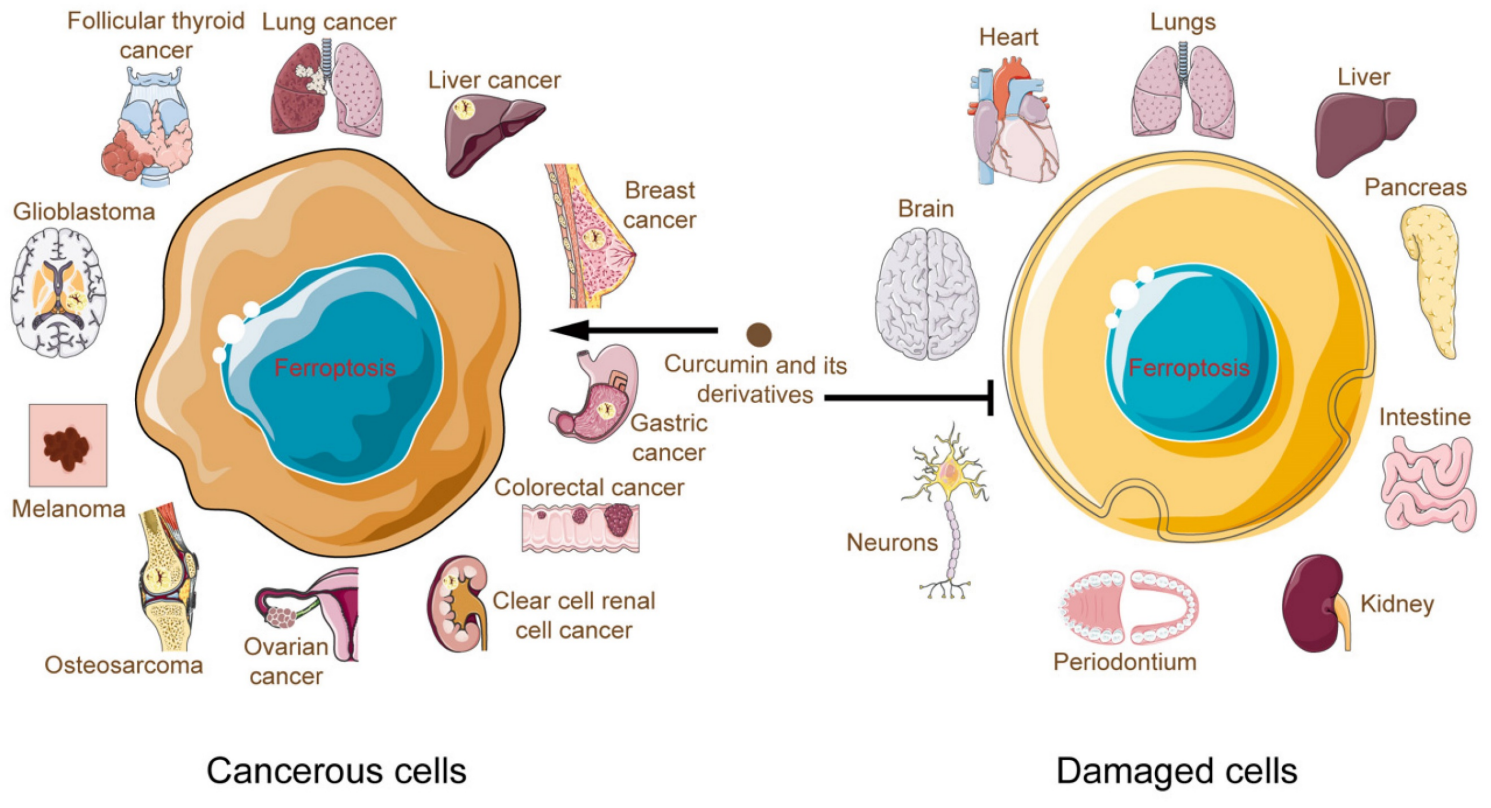
Multifaceted roles of curcumin and its derivatives in ferroptosis as the antiferroptosis or pro-ferroptosis agent.

Curcumin and its derivatives induced death in cancer cells, and bioinformatics analyses have revealed that the ferroptosis pathway was enriched more than other cell death pathways^56^,^105^. Moreover, inhibitors of apoptosis, necrosis, and autophagy failed to counteract this death outcome^48^. This fully justifies the importance of ferroptosis in the process of curcumin potency. For the first time, in this review, we comprehensively summarize the connection between curcumin and ferroptosis. We found that applying curcumin to different disease types and tissues causes ferroptosis to develop differently. In cancer, curcumin, on the one hand, directly or indirectly regulates cellular iron levels and, on the other hand, disrupts the antioxidant system by modulating pathways such as GPX4/GSH, FSP1-CoQ10- NAD(P)H. In contrast, curcumin exerts its iron-chelating effects in noncancer cells and ameliorates oxidative stress, curbing damage associated with ferroptosis in the brain, heart, liver, kidney, and other systems (Fig. 5). These hints point to a complicated curcumin regulation in various cell types that has to be elucidated.

**Figure 6:**
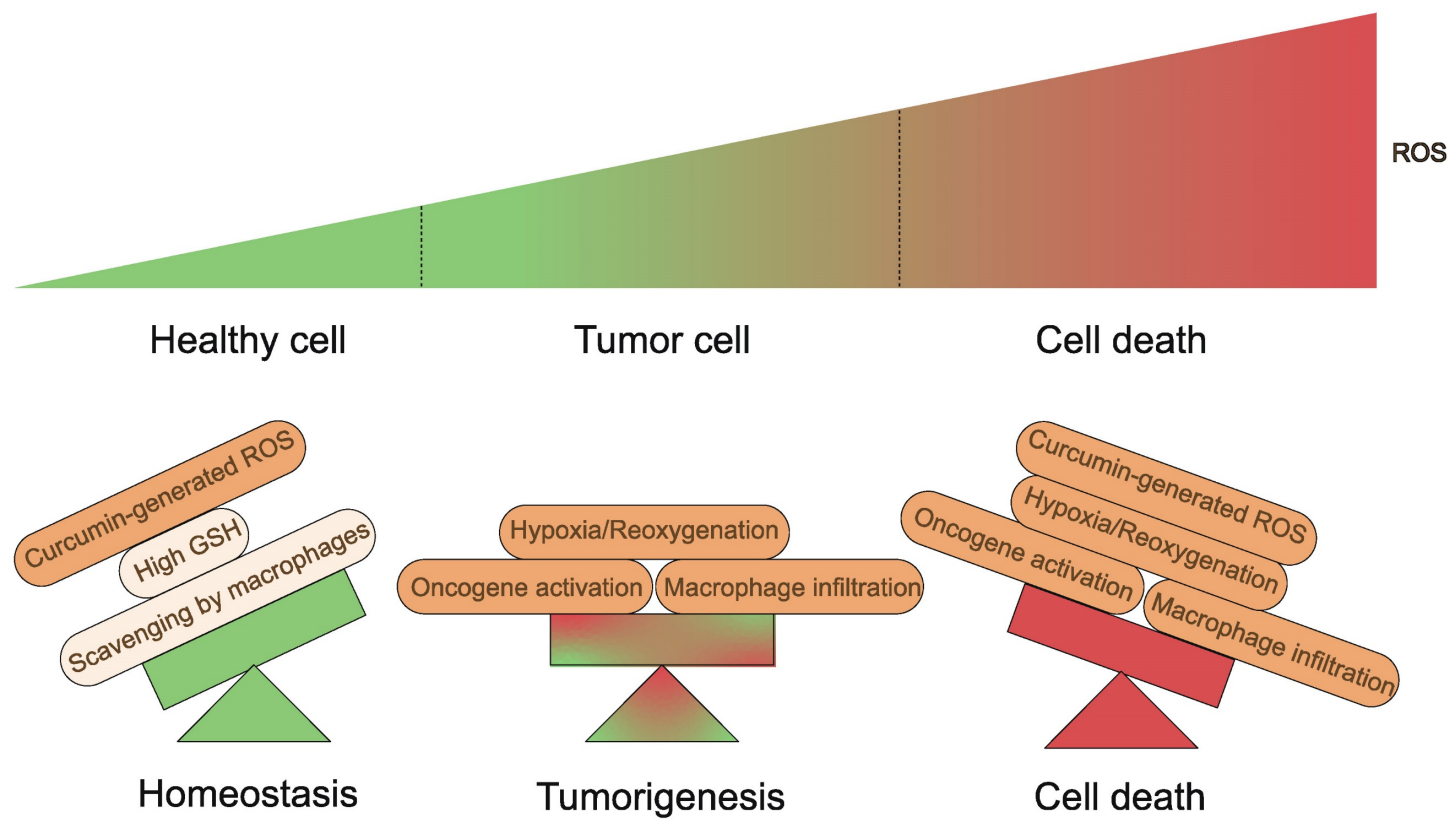
Generation and regulation of cellular ROS. Healthy cells have developed adequate adaptations to overcome the damaging effects of ROS. Balanced generation of ROS, sufficient antioxidant activity and scavenging by macrophages result in low concentrations of ROS. Tumorigenic events including oncogene activation, macrophage infiltration or hypoxia/reoxygenation processes in tissues yield high ROS concentrations. Curcumin-generated ROS become the last straw.

Fundamentally, a variety of elements appear to influence the results produced by curcumin and its derivatives on organisms. To begin with, the TME is a complex system with multiple levels and scales^130^,^131^. Tumor tissues are characterized by different properties than normal tissues, including slightly low pH and ROS overproduction, which lead to cancer cells with intrinsic oxidative stress, which is a key biochemical characteristic that distinguishes cancer cells from normal cells^132^,^133^. Curcumin-generated ROS become the last straw (Fig. 6). Conversely, higher levels of GSH are present in normal cells, which serves as a cellular defense system against ROS^134^. What's more, macrophages are capable of scavenging additional ROS produced in response to curcumin in vivo, thus preventing ferroptosis^83^,^135^. Equally important is that iron in cells is a central factor in cancer progression^136^. Tumor cells contain more iron than normal cells and proteins related to the regulation of iron metabolism are highly expressed in tumor tissues^137^. Therefore, the iron dependence of cancer cells makes them more susceptible to ferroptosis than normal cells^45^,^138^-^142^. Second, at the cellular level, the mechanism of action of curcumin and its derivatives is complex and multifactorial. A recent study found that HO-3867 caused downregulation of p53 in ovarian cancer cells^143^. However, Ling et al. presented experimental results showing that p53 levels tended to increase in NSCLC cells treated with HO-3867^51^. These variations are most likely due to the different doses of curcumin used. Curcumin stimulates HO-1 expression at low concentrations but seems to be less effective at higher concentrations^144^. The hormonal effects of curcumin have also been demonstrated in several studies^145^-^150^. It is a great antioxidant at low doses and has excellent pro-oxidant activity at high doses (≥20 μM)^147^. This also emphasizes the importance of having a proper dosage of the drug in the hands of the clinician.

Natural products, with their wide chemical diversity, have been one of the most valuable avenues for the screening of novel clinical drugs. Izzo et al. demonstrated a new pharmacological practice guideline for the study of natural products, which could be beneficial for the reproducibility of studies on natural products^151^. The majority of research on curcumin presented in this review, however, does not refer to the methodology in the guidelines and suffers from a lack of standardization. In terms of the mechanisms investigated, the FSP1-CoQ10- NAD(P)H pathway is linked to the endosomal sorting complex required for transport III (ESCRT-III), as FSP1 is able to inhibit ferroptosis through a membrane repair process that involves the transportation of the ESCRT-III^152^. Additionally, spermidine/spermine N1-acetyltransferase 1 (SAT1) is a transcriptional target of P53, and activation of SAT1 promotes ROS-induced lipid peroxidation and ferroptosis, which is closely related to the expression of arachidonate lipoxygenase 15 (ALOX-15)^153^. Regrettably, no reports have described curcumin regulation of ferroptosis through modulation of the SAT1, ALOX-15, and ESCRT-III. Likewise, the epigenetic regulation of ferroptosis has been poorly studied. What are the roles of DNA methylation, RNA methylation, and post-translational modifications in the regulation of ferroptosis? How can epigenome editing be used to manipulate tumor cell sensitivity? These questions signal the need for additional pharmacological studies to explore the underlying mechanisms of ferroptosis mediated by curcumin.

## Figures and Tables

**Table 1:** The chemical information of curcumin and some of its derivatives.

Chemical name	Formula	Molecular weight (g/ mol)	Type
Curcumin	C_21_H_20_O_6_	368.38	Natural polyphenol compound
NL01	-	-	-
HO-3867	C_28_H_30_F_2_N_2_O_2_	464.55	Synthesized diarylidenylpieperidone compound
EF24	C_19_H_16_C_l_F_2_NO	347.79	Synthesized monoketone compound
Acetyl zingerone	C_13_H_16_O_4_	236.26	-
MitoCur-1	C_65_H_64_Cl_2_O_6_P_2_	1074.05	-

**Table 2:** Curcumin and its derivatives promoted ferroptosis in cancerous cells.

Disease	Experimental model	Concentration	Major mechanism	Effects	Reference
Glioblastoma	U87MG/A172 cells	0.5-10 μM	AR↓, GPX4↓, ROS↑	Inhibited cell survival	78
Follicular thyroid cancer Lung cancer	FTC-133/FTC-238 cells	1-128 μM	HO-1↑, GPX4↓, MDA↑, GSH↓, ROS↑	Inhibited tumorigenesis	62
	HT29 cells xenograft mice	-	-	Inhibited cell growth, migration and invasion	47
	Lewis cells xenograft mice	100 mg/kg	MDA↑, SOD↓, GSH↓, Fe^2+^↑, ACSL4↑, SLC7A11↑, GPX4↑	Inhibited tumor growth and promoted cell death	49
	A549/H1299 cells	3.1-100 μM	MDA↑, SOD↓, GSH↓, Iron↑, ACSL4↑, SLC7A11↓, GPX4↓	Inhibited cell proliferation and promoted cell death	49
	A549 CD133^+^ cells	0.01-0.08 μmol/ml	ROS↑, GSH↓, CoQ10↓, NAD+/NADH↓, GPX4↓, FSP1↓	Inhibited cellular self-renewal capacity	91
	A549 CD133^+^ cells xenograft mice	100 mg/kg	GPX4↓, FSP1↓	Inhibited tumor growth	91
	H460/PC-9/H1975/A549/H1299/A549 p53 KO/H460 p53 KO cells	5-80 μM	p53↑, DMT1↑, ROS↑, GPX4↓	Inhibited cell viability and promoted cell death	51
	NCI-H460/A549/HepG2/HT-1080 cells	0.5-2 μM	TrxR↓, ROS↑	Promoted cell death	66
	NCI-H460 cells xenograft mice	5-15 mg/kg	TrxR↓, GPX4↓	Inhibited tumor growth	66
	LK-2/H1650 cells	10-40 μM	DMRT3↓, SLC7A11↓	Inhibited cell proliferation, tumorigenesis and induced apoptosis	82
	LK-2 cells xenograft mice	50 mg/kg		Inhibited tumor growth	
Liver cancer	HepG2/HUVECs cells	-	GSH↓	Inhibited cell proliferation and promoted cell death	80
	KMCH/Huh7/PLC cells	25 μM	HO-1↑	Promoted cell death	63
Breast cancer	MCF7/MDA-MB-231 cells	14-50 μM	Nrf2↑, HO-1↑, GPX4↓	Promoted cell death	56
	MCF-7 cells	-	-	Promoted cellular damage	52
	MDA-MB-453/ MCF-7 cells	1-50 μM	ROS↑, MDA↑, Fe^2+^↑, SLC1A5↑, GPX4↓, FTL↓, ACSL4↑, NOX1↑	Promoted cell death	50
	MCF-7 cells xenograft mice	30 mg/kg	MDA↑, Fe^2+^↑, SLC1A5↑, GSH↓	Inhibited tumorigenesis	50
	MCF-7/MDA-MB-231 cells	5-50 μM	HO-1↑, GPX4↓, FHC↑, Fe^2+^↑, LOOH↑	Inhibited cell viability	58
Gastric cancer	-	-	TLR4, KRAS	-	105
Colorectal cancer	HCT-8 cells	1-100 μM	Iron↑, MDA↑, ROS↑, GSH↓, GPX4↓, SLC7A11↓, p-PI3K↓, p-Akt↓, p-mTOR↓	Inhibited cell proliferation	83
	SW480/HCT116 cells	1-5 μg/ml	GPX4↓, FSP-1↓	Inhibited cell proliferation, clone formation and induced apoptosis	92
	SW620/LoVo cells	10-80 μM	p53↑, GPX4↓, SLC7A11↓	Inhibited cell proliferation, migration and clone formation	84
	SW620 cells xenograft mice	100 mg/kg		Inhibited tumor proliferation	
	SW480 cells	0-100 μM	JNK↓	Inhibited cell proliferation	100
	SW480 cells	5-50 μM	-	Inhibited cell proliferation	101
Clear cell renal cell cancer	A498/786-O cells	2-10 μM	ADAMTS18↑, NCOA4↓, FTH1↓, p53↓	Inhibited cell proliferation	57
Ovarian cancer	Anglne/HO8910PM cells	1-8 μM	HCAR1↓, MCT1↓	Inhibited cell growth	106
	HO8910PM cells xenograft mice	5 mg/kg		Inhibited tumor proliferation	106
Osteosarcoma	U2os/Saos-2 cells	0.5-4 μM	HO-1↑, GPX4↓, MDA↑, ROS↑, Iron↑	Inhibited cell viability and promoted cell death	48
	MNNG/HOS/MG-63 cells	-	Nrf2↓, GPX4↓	Inhibited cell proliferation and invasion, induced apoptosis and G0/G1 phase arrest	79
	MNNG/HOS xenograft mice	-		Inhibited tumor proliferation	
Melanoma	A375/B16 cells	-	LPO↑	Promoted cell death	81
	A375/SKMEL28 cells	1-4 μM	USP14↓, GPX4↓, SLC7A11↓, GSH↓	Inhibited cell proliferation and migration, induced apoptosis and cell cycle arrest	102

**Table 3:** Curcumin and its derivatives inhibited ferroptosis in tissue-damaged models.

Disease	Experimental model	Concent-ration	Major mechanism	Effects	Reference
Diabetic neuropathy	N2a cells	0.005 μmol/ ml	Fe^2+^↓, GPX4↑, SLC7A11↑, FTH1↑, TFR-1↓, Nrf2↑, HO-1↑	Inhibited nerve cell death and promoted nerve cell viability	108
Intracerebral hemorrhage	ICH rats	0.001-100 μM	Nrf2↑, HO-1↑	Promoted the clearance of intracranial hematoma, reduced perihematomal brain edema, and promoted the recovery of neurological functions	109
	Neurons	10 μM	Fe^2+^↓, Iron↓, GPX4↑, FTH1↑, SLC7A11↑, ACSL4↓	Reduced cell damage and nerve death	110
	ICH rats	-		Reduced blood-brain barrier dysfunction in brain tissue surrounding hematoma	110
	HT22 cells	2.5-320 μM	ROS↓, Nrf2↑, HO-1↑	Inhibited hippocampal cell death	111
Neurodegenerative disorders	HT22 cells	10-50 μM	ARE↑, HO-1↑	Protected nerves	112
		10-25 μM	GCLC↑, Sp1↑	Promoted neuronal survival	113
		0.1-10 μM	ROS↓, Fe^2+^↓	Inhibitd oxidative apoptosis and protected dopaminergic neurons	114
		0.1-10 μM	ROS↓	Inhibited hippocampal cell death	115
Valvular atrial fibrillation	HL-1 cells	0.005-1 μmol/ ml	TGFBR1↓	Reduced myocardial fibrosis	116
Dabetic cardiomyopat-hy	Diabetic rabbits	300 mg/kg	-	Improved myocardial structure	117
	H9C2 cells	0.001-0.018 μmol/ ml	Nrf2↑, HO-1↑, GPX4↑	Alleviated the injury of cardiac myocytes and reversed the death of cardiac myocytes	117
Ischemia/repe-rfusion injury	Ischemia/repe-rfusion-damaged rats	100 mg/kg	ACSL↓, GPX4↑	Reduced damage to the heart, liver and pancreas	118
Hepatolenticul-ar degeneration	TX mice	50-100 mg/kg	-	Inhibited liver damage	119
	BRL-3A cellls	2.5-10 μM	Nrf2↑, HO-1↑, GPX4↑		
Liver injury	Gibel carp with liver injury	-	ROS↓, MDA↓, Nrf2↑, ACSL4↓, PTGS2↓, SLC7A11↑	Improved mitochondrial morphology	120
Acute kidney injury	Mice with rhabdomyolys-is	1000 mg/kg	HO-1↑, MDA↓, GSH↑	Improved the function and histology of renal damage	122
	MCTs/HK-2 cells	10 μM			
	HK-2 cells	1-40 μg/ml	ROS↓, Iron↓	Improved renal function and reversed the pathological process of IR-AKI	123
	Human renal tubular epithelial cells	5-40 μM	p62↑, Keap1↑, Nrf2↑	Promoted cell proliferation	121
	Mice with kidney injury	50 mg/kg		Reduce the histopathological lesions in the kidney	
	Ducks with kidney injury	400 mg/kg	NCOA4↓	Alleviated growth retardation and renal distorted structure	41
COPD	BEAS-2B cells	5-20 μM	MDA↓, Iron↓, ROS↓, GSH↑, SLC7A11↑, GPX4↑, FTH1↑, TFR1↓	Improved lung injury and inflammation	124
	Rats with lung epithelial injury	100 mg/kg	MDA↓, Iron↓, SLC7A11↑, GPX4↑, FTH1↑, TFR1↓		
Inflammatory bowel disease	IEC-6 cells	0.125/ 1.25 μM	GSH↑, GPX4↑, MDA↓	Improved mitochondrial morphology	125
	IBD mice	4 mg/kg		Improved typical features of ulcerative colitis, restored the histological structure of the colon, and reduced the destruction of colonic tissue	125
Periodontitis	Mice with periodontitis	50-200 mg/kg	SOD↓, GSH↑, MDA↓, LC7A11↑, GPX4↑, ACSL4↓, TfR1↓	Reduced periodontal tissue damage	126
-	MIN6 pancreatic cells	5-20 μM	Iron↓, MDA↓, GSH↑, GPX4↑	Inhibited MIN6 cell death	107
Osteoarthritis	Mouse chondrocytes	0.5-32 μM	Nrf2↑	Promoted cell proliferation	127
	Knee OA mice	50 mg/kg		Attenuated cartilage degeneration, cartilage erosion and matrix los	
	Rat chondrocytes	20-100 μM	GPX4↑	Inhibited apoptosis	128
	Knee OA mice	0.5-1 mg/kg/ body weight	Nrf2↑, HO-1↑	Attenuated articular cartilage degeneration	
Testicular damage	Leydig/sertoli cells	10-30 μM	SP1↑, PRDX6↑	-	129
	Rat with testicular damage	300 mg/kg		Attenuated testicular damage	

## Abbreviations

AIFM2: apoptosis-inducing factor mitochondria-associated protein 2
Akt: protein kinase B
ALOX-15: arachidonate lipoxygenase 15
AMPK: AMP-activated protein kinase
CAT: catalase
COPD: chronic obstructive pulmonary disease
CoQ10: coenzyme Q10
DMT1: recombinant divalent metal transporter 1
ECM: extracellular matrix
ESCRT-III: endosomal sorting complex required for transport III
FDA: Food and Drug Administration
FSP1: ferroptosis suppressor protein 1
FT: ferritin
FTH1: ferritin heavy chain
FTL: ferritin light chain
Gox: glucose oxidase
GPX: glutathione peroxidase
GPX4: glutathione peroxidase 4
GSH: glutathione
GSSG: oxidized glutathione
HCAR1: hydroxycarboxylic acid receptor 1
HLD: hepatolenticular degeneration
HO-1: heme oxygenase 1
IBD: inflammatory bowel disease
ICH: intracerebral hemorrhage
IR-AKI: ischemia-reperfusion acute kidney injury
IREB2: iron-responsive element binding protein 2
JNK: c-Jun N-terminal kinase
KRAS: Kirsten rat sarcoma viral oncogene
LPO: lipid peroxide
L-ROS: lipid reactive oxygen species
MCT1: monocarboxylic acid transporter protein 1
MDA: malondialdehyde
mTOR: mechanistic target of rapamycin
NAD(P)H: nicotinamide adenine dinucleotide phosphate hydrogen
NCOA4: nuclear receptor coactivator 4
Nrf2: nuclear factor-E2-related factor 2
OA: Osteoarthritis
OM-MSCs: mesenchymal stem cells
p53: transformation related protein 53
PAAs: pro-oxidant anticancer agents
PI3K: phosphatidylinositol 3-kinase
PRDX6: peroxiredoxin 6
PUFA: polyunsaturated fatty acid
PUFA-PLs: polyunsaturated fatty acid-containing phospholipids
REBP1: sterol regulatory element-binding protein 1
ROS: reactive oxygen species
SAT1: spermidine/spermine N1-acetyltransferase 1
SLC1A5: recombinant solute carrier family 1, member 5
SLC3A2: recombinant solute carrier family 3, member 2
SLC7A11: recombinant solute carrier family 7, member 11
SOD: superoxide dismutase
SP1: specific protein 1
TF: transferrin
TFR1: transferrin receptor 1
TGFBR1: transforming growth factor beta receptor 1
TLR4: toll-like receptor 4
TME: tumor microenvironment
Trx: thioredoxin
TrxR: thioredoxin reductase
USP14: ubiquitinspecific protease 14
